# The prevalence of stroke among adults in Ethiopia from 2012 to 2022: A systematic review and meta-analysis protocol

**DOI:** 10.1371/journal.pone.0285678

**Published:** 2023-05-17

**Authors:** Maycas Dembelu, Teklu Wosenyeleh, Wubishet Gezimu, Diriba Kumara

**Affiliations:** 1 Department of Nursing, College of Health Science, Mettu University, Mettu, Ethiopia; 2 Department of Health Informatics, College of Health Science, Mettu University, Mettu, Ethiopia; SPHMMC: St Paul’s Hospital Millennium Medical College, ETHIOPIA

## Abstract

**Introduction:**

Stroke is one of the most common causes of death and acquired disability worldwide. The burden of death and disability-adjusted life-years lost (DALYs) in lower-and-middle income countries (LMIC), were 86% and 89%, respectively. Ethiopia, one of SSA countries, is being affected by stroke and its consequence. The conception and development of this systematic review and meta-analysis protocol primarily based on the gap we noted from the previous systematic review and meta-analysis. Thus, this review will fill knowledge gap by identifying and analyzing studies that used sound method in determiningthe last ten years stroke prevalence in Ethiopia.

**Methods:**

This systematic review and meta-analysis will follow Preferred Reporting Items for Systematic Reviews and Meta-analyses guideline. Both published articles and gray literature will be gathered from online databases. Cross-sectional, case control, and cohort studies will be included as long as these studies report the magnitude of the problem under study. Both community and facility-based studies conducted in Ethiopia will be included. Those studies that did not report the main outcome variable will be excluded. Joanna Bridge Institute appraisal checklist will be used to assess the quality of individual studies. Two reviewers will independently appraise the full articles of studies related to our topic of interest. I^2^ and p-value will be used to check for heterogeneity of the studies’ outcome. Meta-regression will be used to identify source of heterogeneity. We will assess the presence of publication bias using funnel plot.

**PROSPERO registration number:** CRD42022380945.

## Introduction

Stroke is one of the most common causes of death and acquired disability worldwide [1]; according to Global Burden of Disease (GBD) report, stroke was the second leading cause of death and the third leading cause of death and disability worldwide [2]. Globally, five and a half-million people die from stroke annually, and over 116 million years of healthy life lost (YLL) each year due to stroke related death and disability [3]. The burden of death and disability-adjusted life-years lost (DALYs) in lower-and-middle income countries (LMICs), were 86% and 89%, respectively. In LMIC, particularly in sub-Saharan countries, there was high stroke associated mortality (15-fold), prevalence (4-fold), and disability adjusted life years (DALYs) (20-fold) rates [2, 4].

The vast majority of stroke cases can be categorized into two major classes: Ischemic stroke and hemorrhagic stroke. Ischemic stroke is caused by blockage of artery in the cerebral vascular area while hemorrhagic stroke is caused by rupture of blood vessel in the cerebral vascular area. Consequently, both types of strokes cause cerebral hypoxemia that damage brain cells [5–7]. Metabolic risks (high systolic blood pressure (SBP), high body mass index (BMI), high fasting plasma glucose (FPG), high total cholesterol, and low glomerular filtration rate) accounted for 72.1% (66.4–77.3) of stroke burden [3]. Factors like age, gender, ethnicity, and heredity were also identified as determinant for stroke occurrence [3, 7, 8].

Stroke is associated with disability (as expressed by DALY) as its burden. Stroke is the second most contributor of DALY next to ischemic heart disease globally and in developing countries. Due to change in life style in LMICs, stroke associated incidence, disability, and death has increased globally. Since stroke is a vascular disease, death due to stroke, is the other public health problem affecting millions worldwide. Hemorrhagic stroke, one type of stroke, accounted for 80% of mortality rate in low-and-middle income countries [9]. Based on a report from systematic review and meta-analysis in sub-Saharan Africa (SSA) countries, the 5 year case fatality rate was around 40%. Again, this study identified that individuals with diabetes had poor prognosis [10].

Ethiopia, one of SSA countries, is being affected by stroke and its consequence. As Ethiopia is a country with poor socio-economic condition, in recent decades, resource has been dedicated to communicable disease control programs; but enough attention has not been given to stroke and other non-communicable disease (NCD).As a result, stroke has emerged as one of public health concern in recent years [11, 12]. Several studies have been conducted concerning stroke across different regions of Ethiopia [13–15]. One systematic review and meta-analysis tried to identify the burden of stroke in Ethiopia [16]. According to this systematic review and meta-analysis, the burden of ischemic and hemorrhagic stroke were 51.40% and 46.42%, respectively. The conception and development of this systematic review and meta-analysis protocol primarily based on the gap we noted from the previous systematic review and meta-analysis. The former systematic review and meta-analysis used primary studies that were not methodologically appropriate to show the burden of stroke. Some of the primary studies, which were included in the previous systematic review and meta-analysis, used stroke patients as a denominator (i.e. Stroke was not their outcome variable). So, the previous systematic review and meta-analysis had limitation in identifying source population for stroke cases, which affects its generalizability. Thus, this study will fill knowledge gap by identifying and analyzing studies that used sound method in determining the last ten years stroke prevalence in Ethiopia.

## Review question or objective of systematic review and meta-analysis

Since this review will be conducted based on prevalence of stroke assumption, our review question includes the following terms:

-population = all adult population in Ethiopia

-outcome = all adult stroke cases in Ethiopia

This systematic review and meta-analysis will report ten year burden of stroke among adult individuals in Ethiopia.

## Method

### Review registration and report of the systematic review

This protocol has been registered in the international prospective register of systematic reviews and meta-analysis (PROSPERO) and the PROSPERO registration number is CRD42022380945.Development, conduct, and reporting of this systematic review and meta-analysis will follow Preferred Reporting Items for Systematic Reviews and Meta-analyses (PRISMA) guideline [17].

### Data source and search strategy

Both published articles and gray literature will be gathered from the following databases: PubMed, science direct, Cochrane library, Google scholar, Medline, and local universities’ databases. We will also look for other relevant literature from the reference of identified articles, and manual search will be performed to get access to additional literature. If we encounter difficulty in accessing the full article, we will contact the corresponding author through email. We will use all field and medical subject heading (MeSH) terms in PubMed database search; Boolean operators (OR, AND, and NOT) will be used to join key terms. The following terms will be used for identifying literature: “stroke”, “stroke status”, “cerebral vascular hemorrhage” “magnitude”, “prevalence”, “Ethiopia”. Database and manual search will be employed for studies that will be published until March 20, 2023.

### Inclusion and exclusion criteria

Observational studies including cross-sectional, case-control, and cohort studies will be included as long as these studies report the magnitude of the problem under study. All adult 18 years old and above stroke patients will be included in this systematic review and meta-analysis. Both community and facility-based studies conducted in Ethiopia will be included. Studies conducted within the last ten years will also be included. Those studies that did not report stroke as the main outcome variable will be excluded. Furthermore, those studies that the author of this systematic review and meta-analysis unable to find the full article will be excluded.

### Study selection and data extraction

First, titles and abstracts of studies will be reviewed against our predetermined inclusion and exclusion criteria and duplicate studies will be removed. Mendeley reference manager software will be used to remove the duplicate article, after comparing the content of the duplicate studies with the main study. Then, the full text of the remaining studies will be retrieved and appraised for inclusion in our systematic review. Finally, study selection and data extraction will be done. Check list that shows both selection criteria and included and excluded studies will be prepared in the form of a table. The whole process of study selection will be reported on PRISMA flow chart.

To extract data, excel spreadsheet will be prepared. WG and DK will extract data. MD and TW will follow the data extraction process. Pertinent information like author’s name, year of publication, sample size, number of individuals with the outcome (stroke), study design, study type (community or facility based), study area (region) will be extracted into the excel sheet.

### Quality assessment

Joanna Bridge Institute (JBI) appraisal checklist [18] will be used to assess the quality of individual studies. Two reviewers (MD and TW) will independently appraise the full articles of studies related to our topic of interest. If there is disagreement between the two reviewers, an experienced colleague will be invited to resolve issues in the appraisal process.

### Data analysis

After data extraction into excel spread sheet, data will be exported to STATA v.14 statistical software for analysis. The pooled prevalence of included studies will be computed. Summary statistics of individual studies will be presented in a graph. Cochrane’s Q statistic, I^2^ and p-value will be used to check heterogeneity of the studies’ outcome. I^2^ of 25%, 50%, and 75% will be used as an indicator for low, moderate, and high heterogeneity, respectively [19]; forest plot will be used to visualize heterogeneity. If there is moderate to high heterogeneity, random effect meta-analysis will be employed. Meta-regression will be used to identify source of heterogeneity. A statistical significant result from Meta-regression will be declared as a source of heterogeneity. We will perform subgroup analysis in order to identify variables for heterogeneity. We will assess the presence of publication bias using funnel plot; a statistical significance result from Egger and Begg tests will also be used as an indicator of publication bias. Leave-one-out sensitivity analysis will be performed to assess single study effect.

### Ethics and dissemination

Since there is no involvement of human study participants, ethical approval will not be required to conduct this systematic review and meta-analysis. The result of this study will be reported in internationally reputable journals, and it will also be presented at different research conferences.

## Supporting information

S1 Checklist PRISMA-P (Preferred Reporting Items for Systematic review and Meta-Analysis Protocols) 2015 checklist: Recommended items to address in a systematic review protocol*. (DOC)
